# Down-regulation of PADI2 prevents proliferation and epithelial-mesenchymal transition in ovarian cancer through inhibiting JAK2/STAT3 pathway in vitro and in vivo, alone or in combination with Olaparib

**DOI:** 10.1186/s12967-020-02528-0

**Published:** 2020-09-20

**Authors:** Lidong Liu, Zhiwei Zhang, Guoxiang Zhang, Ting Wang, Yingchun Ma, Wei Guo

**Affiliations:** 1 Department of Obstetrics and Gynecology, The First Affiliated Hospital of Shandong First Medical University, Jinan, 250014 Shandong People’s Republic of China; 2 Medical Research Center, The First Affiliated Hospital of Shandong First Medical University, Jinan, 250014 Shandong People’s Republic of China; 3 Obstetrics Department, Obstetrics and Gynecology Hospital of Fudan University, Shanghai, 200011 People’s Republic of China

**Keywords:** PADI2, Epithelial ovarian cancer, Olaparib, Epithelial-mesenchymal transition, JAK2/STAT3 pathway

## Abstract

**Background:**

Epithelial ovarian cancer (EOC) is the most lethal disease among female genital malignant tumors. Peptidylarginine deiminase type II(PADI II) has been shown to enhance a variety of cancers carcinogenesis, including ovarian cancer. The purpose of this study was to investigate the biological role of PADI2 in ovarian cancer (OC) and the relative mechanism.

**Methods:**

Gene Expression Profiling Interactive Analysis (GEPIA) (https://gepia.pku.cn/) and ONCOMINE (https://www.oncomine.org/) were used to analyze PADI2 Gene Expression data. The survival curve for the PADI2 gene was generated by using the online Kaplan–Meier mapping site (https://www.kmplot.com/). We conducted MTT assay, cloning formation assay and EdU cell proliferation assay to detect the cell activity of PADI2 knockdown A2780 and SKOV3 ovarian cancer cells treated with Olaparib. Cell migration and invasion were observed by would healing and transwell assay. The pathway changes after the treatment of PADI2 were detected by transcriptome sequencing and western blot. The role of PADI2 combined with Olaparib treatment in vivo was studied in nude mouse model bearing ovarian cancer tumor.

**Results:**

We investigated the role of PADI2 on EOC in vitro and in vivo. PADI2 was upregulated in ovarian cancer samples and high PADI2 expression was correlated with poor outcome. Downregulating PADI2 suppressed colony formation, proliferation, migration and invasion of A2780 and SKOV3 cells. Furthermore, downregulating PADI2 and Olaparib combination treatment attenuated the viability, migration and invasion of A2780 and SKOV3 cells. We identified differentially expressed genes in A2780-shPADI2 and SKOV3-shPADI2 cell by transcriptome sequencing analysis and verified that downregulating PADI2 and Olaparib combination treatment suppresses EMT and JAK2/STAT3 signaling pathway in A2780 and SKOV3 cells in vitro and in vivo.

**Conclusions:**

Downregulation of PADI2 and Olaparib combination treatment attenuated the proliferation, migration and invasion of A2780 and SKOV3 cells by inhibiting the EMT through JAK2/STAT3 signaling pathway.

## Background

Epithelial ovarian cancer (EOC) is the most lethal disease among female genital malignant tumors [1]. American cancer statistics in 2020 showed that 13,940 of 21,750 new ovarian cancer patients will die [2].Ovarian cancer is insidious, with more than two-thirds of patients showing symptoms at advanced stage. Cytoreductive surgery and chemotherapy based on platinum plus paclitaxel drugs are the main treatment strategies for advanced ovarian cancer. However, about 70% of patients relapse within 3 years and eventually develop distant metastasis [3]. Although the emergence of a PARP inhibitor, Olaparib, currently used as a maintenance treatment for ovarian cancer, represented a major breakthrough in cancer treatment, resistance has emerged [4, 5].PARP inhibitors initially show good clinical response, but most patients develop resistance to these drugs [6–8]. Therefore, it is necessary to understand the molecular mechanism of the occurrence, development and drug resistance of ovarian cancer deeply to find new targets that can inhibit the drug resistance of ovarian cancer, so as to improve the efficiency of the treatment of ovarian cancer. Further exploration of the mechanism of the development and metastasis of OC and the provision of therapeutic targets is urgently required.

Peptidylarginine deiminase (PAD) is a post-translational modification enzyme that converts positive charged arginine into neutral charged citrulline. PAD has been shown to have a wide range of effects on target protein structure, function, and protein–protein interactions. A growing number of reports have linked PAD disorder to a range of diseases, including rheumatoid arthritis, multiple sclerosis, ulcerative colitis, COPD and cancer [9–11]. Although PAD's function in most diseases is associated with inflammation, the role of PADI in cancer progression is still being investigated [11–14]. Expression of Peptidylarginine deiminase type II (PADI II) has been shown to enhance a variety of cancers carcinogenesis, including breast cancer [15], spontaneous skin tumors [16], epithelial bladder cancer [17] and colon cancer [18]. McElwee et al. [19]found that PADI2 was a potential biomarker and therapeutic target for breast cancer and found that transgenic mice with overexpression of PADI2 were prone to spontaneous skin neoplasia [20]. Tanday [21] reported that inhibiting the expression of PADI2 prevented the progression of myeloma. It has also been reported that PADI2 is a breast cancer susceptibility gene, whose expression promotes tumor development through ACSL4, BINC3 and CA9 signaling pathways [15]. These results suggest that PADI2 is involved in the development of many tumors and plays an important role in the progression, while the mechanism of PADI2 in ovarian cancer has not been reported.

Epithelial mesenchymal transformation (EMT) is a process in which polar epithelial cells transform into transitional mesenchymal cells and acquire the ability to invade and migrate. EMT is a process of phenotypic plasticity that entrusts epithelial cells with migration and invasiveness during development, wound healing, fibrosis, and cancer, and is jointly driven by SNAIL, ZEB, and TWIST transcription factors [22]. EMT is a multi-step dynamic change process. The cells in the center of solid tumors are epithelial cell phenotype, and the cells around them often show mesenchymal cell phenotype. With its strong movement ability, tumor cells can locally infiltrate and invade blood and lymphatic vessels to metastasize to the target organs. EMT is closely related to drug resistance to tumor metastasis. Chowdhury et al. [23]reported bone sarcoma cell line U2OS, human non-small cell lung cancer cell line A549 and human cervical cancer cell line HeLa three different human cancer cells, the gamma rays joint PARP inhibitors olaparib illuminate, epithelial-interstitial cell migration, transformation pathways involved in the change some marker protein expression and activity, which alone olaparib decreased cell migration in vitro and at the same time reduce the N—cadherin and vimentin EMT pathway marker protein expression, etc. In another of his reports [24], two non-small cell lung cancer A549 and p53-deficient H1299 cells were irradiated with ^12^C and inhibited by PARP-1 to reduce cell proliferation and cell migration, respectively. Among them, single treatment reduced n-cadherin and Vimentin, but increased claudin-1 and -2 to inhibit EMT. Schack et al. [25]reported that Olaparib prevented EMT from occurring. Furthermore, Chen et al. [26]found that PADI2 was closely related to the process of EMT. Therefore, we hypothesized that targeting PADI2 could affect the treatment of ovarian cancer with Olaparib through EMT.

Janus kinase (JAK)/signal transductor and activator of transcription (STAT) signaling pathways are downstream pathways of cytokine signaling, regulating cell development, differentiation, proliferation, and apoptosis, which not only participate in regulating normal physiological processes, but also play an important role in the occurrence and development of tumors [27, 28]. Now it has been reported [16] that targeting PADI2 in cancer is associated with phosphorylated STAT3. Furthermore, multiple reports [29, 30] have shown that Olaparib is associated with JAK2 and STAT3 genes and inhibits STAT3 phosphorylation. The expression of PADI2 was up-regulated in NNK [4-(methylnitrosamino)-1-(3-pyridyl)-1butanone]-treated lung tissues and played a substantial role in the induction of lung cancer through STAT3 pathway [31].

Altogether, In our previous study, immunohistochemistry and ELLSA were used to detect a significant increase in the expression of PADI2 in cervical squamous cell carcinoma, ovarian serous papillary adenocarcinoma, etc. [32]. Therefore, in this study we investigated the role of PADI2 on EOC in vitro and in vivo for the first time and further determined whether PADI2 knockdown could inhibit invasion and migration in Olaparib resistance to EOC by inhibiting EMT by targeting JAK2 / STAT3 signaling pathway in vitro and in vivo. Therefore, in this study we provided a novel manipulating strategy for Olaparib resistance to ovarian cancer by downregulation of PADI2.

## Methods

### Cells and reagents

The human ovarian cancer cell lines A2780 and SKOV3 cells were purchased from The American Type Culture Collection (ATCC). Olaparib (AZD2281) were purchased from MedChemExpress (Shanghai, China). Dimethyl sulfoxide (DMSO) was obtained from Solarbio (Beijing, People’s Republic of China). The antibodies against E-cadherin (3195S), Vimentin (5741S), and GAPDH (2118S) were purchased from Cell Signaling Technology (CST, MA, USA).

### Cell culture and siRNA interference

A2780 and SKOV3 cells were cultured in Roswell Park Memorial Institute-1640 (RPMI-1640) medium(Gibco; Thermo Fisher Scientific, Inc.), supplemented with 10% fetal bovine serum (FBS, Biological Industries, Kibbutz Beit Haemek, Israel). Cells were in an incubator of 5% CO2 at 37 °C. The medium was replaced every 2 days. The siRNA oligonucleotides targeting the PADI2 gene were commercially obtained from Shanghai GenePharma Co., Ltd. A2780 and SKOV3 cells were transfected with the anti-PADI2 siRNAs using Lipofectamine® 2000 (Invitrogen; Thermo Fisher Scientific, Inc.) for 48 h. The inhibition of PADI2 expression in these cell lines was verified using Western blot.

### Lentivirus production and infection

PADI2 short hairpin RNA (shRNA) was purchased from GenePharma company (Shanghai, China). A2780 and SKOV3 ovarian cancer cells at logarithmic growth stage were inoculated into a 6-well plate overnight. On the second day, 500 μL PADI2 shRNA virus was added into each well and cultured in a constant temperature incubator for 6 h. Then, A2780 and SKOV3 ovarian cancer cells were successfully transfected into a new complete culture medium with 2 μg/ml puromycin (Merck Millipore, Billerica, MA, USA) to acquire stable expression cells selecting for 2 weeks.

### Cell viability and proliferation assays

Cells were seeded into a 96-well plate at a density of 3000 cells/well. Cell viability was measured by Methylthiazolyl tetrazolium(MTT) (Solarbio, Beijing, People’s Republic of China). In brief, after cells treated with olaparib for 48 h, 10 μL MTT (5 g/L) solution was added to each well, incubated for 4 h at 37 °C in humidified 5% CO2 and 95% air atmosphere and dissolved in 100 μL DMSO per well. The absorbance at 490 nm wave length was evaluated by Microplate Reader (Bio-Rad, Hercules, CA, USA). 5-ethynyl-2′-deoxyuridine (EdU) (Ribobio, Guangzhou, China) incorporation assay was used to access proliferation according to the manufacturer’s protocol. Cell nuclei were stained with DAPI for 15 min and then observed under a fluorescence microscope (Leika DMi8,Germany).

### Clonogenic assay

Cells were seeded on 6 well plates and incubated overnight. After treatment of Olaparib, complete growth medium was replaced every 3 days. Finally, cells were fixed with Paraformaldehyde and stained with 5% crystal violet solution. Images of stained plates were photographed and analyzed.

### Transwell migration and invasion assays

Transwell migration and invasion assays were performed with 24-well Boyden chambers (Corning Costar, Cambridge, MA, USA). Cells were re-suspended in 100μL serum-free medium (2 × 10^4^ SKOV3 cells or 5 × 10^4^ A2780 cells) and then seeded onto the top of the chamber in 100 μL serum free medium. The lower chamber was filled with 750 μL medium containing 10% FBS. After incubation for 24 h, the cells that had migrated, on the lower surface of the filter, were fixed with methanol solution for 5 min and 4% paraformaldehyde for 5 min and then stained with Giemsa stain. Images (200 ×) of stained cells were captured with an Olympus X71 inverted microscope (Olympus Corporation, Tokyo, Japan). Cells were counted in five visual fields per chamber. For the invasion assay, Boyden chambers were pre-coated with 100 μL Matrigel (1:9 dilution in serum free medium; Beijing Solarbio Science & Technology Co., Ltd.) prior to experimentation. Following this, similar steps were performed as in the migration assay.

### Wound healing assay

After A2780 and SKOV3 cells were transfected with the anti-PADI2 siRNAs, 10 × 10^4^ cells per well were seeded in 6‐well plates and incubated overnight. Wounds were scratched using 1 mL pipette tips. Cells reached confluence and were photographed at 0, 24, 48, 72 h points.

### Western blot analysis

A2780 and SKOV3 cells were harvested, lysed in a mixed buffer contained RIPA, NaF and PMSF (100:1:1) and centrifuged at 12,000 rpm for 15 min after the indicated treatments. The supernatants were separated, mixed with 5 × loading buffer and boiled for 5 min. Proteins from each sample were separated by 12% SDS-PAGE and transferred to PVDF membranes (Immobilon-P; Millipore, USA). Then, membranes were blocked in 5% non-fat milk at room temperature for 2 h, incubated with primary antibodies (1:1000) overnight at 4 °C, washed with Tris-Buffered Saline with Tween-20 and incubated with HRP-linked anti-rabbit IgG secondary antibodies (1:3000) at room temperature for 1 h. Proteins were detected by Chemiluminescent HRP Substrate (Merck Millipore, Billerica, MA, USA) and observed by FluorChem M biomolecular imager (proteinsimple, US). The gray values were analyzed by ImageJ software.

### Survival analysis

Kaplan–Meier curves for target genes were generated with the online tool Kaplan–Meier Plotter(https://www.kmplot.com/). The RNAseq data samples of ovarian cancer patients were split into 2 groups based on the expression level. We analyzed the progression-free survival (PFS) of ovarian cancer patients stratified by high and low expression of PADI2 using the Kaplan–Meier curves, the log rank test and the hazard ratio with 95% confidence intervals (CI).

### Gene expression data

Gene expression data were obtained from Gene expression profiling interaction analysis(GEPIA) (https://gepia.cancer-pku.cn/) and ONCOMINE website (https://www.oncomine.org/). Cancer type was restricted by ovarian cancer, and the expressions of PADI2 were obtained. In our study, we utilized online tools to analyze the levels of PADI2 between ovarian cancer specimens and normal controls.

### Transcriptome sequencing

After total RNA concentration was measured, library construction and Illumina platform sequencing were carried out by biological company to obtain original reads. Clean Reads were obtained through data processing, such as decontamination. DESeq2 software was used to analyze the differential expression of the obtained mRNA and screen out up-regulated and down-regulated expression genes. After filtering and quality assessment of the original sequencing data, differentially expressed genes caused by PADI2 gene changes were screened out.

### Tumor xenograft

4 weeks female nude mice (BALB/c) were obtained from Vitalriver (Beijing, China) and raised in specific pathogen-free (SPF) conditions. Each nude mouse was injected subcutaneously with 200 μL PBS suspended of SKOV-3 cells (1 × 10^7^ cells). 20 nude mice were randomized into 4 groups (n = 5). The mice in indicated groups were injected intraperitoneally with Olaparib (50 mg/kg) every day for 21 days. The volume (V = 1/2 × length × width^2^) of subcutaneous tumors and body weight of each mouse were measured every three days. These nude mice were sacrificed by carbon dioxide asphyxiation as indication. Tumor masses from the mice were dissected, weighed and fixed in 4% paraformaldehyde for immunohistochemistry staining. All of procedures were admitted by Experimental Animal Ethics Committee of the First Affiliated Hospital of Shandong First Medical University.

### Immunohistochemistry

Tumor masses were fixed in 4% paraformaldehyde for at least 2 days and embedded in paraffin. Tumor Sects. (3 μm thickness) were cut from the paraffins by a microtome and dried at 60 °C for at least 2 h. Then, the sections were deparaffinized in xylene and incubated in gradient concentrations of ethanol and 10 mM citrate buffer (pH 6.0) at 95 °C for 2 min to perform antigen retrieval. After cooling to room temperature, the samples were incubated in 3% H_2_O_2_ solution for 20 min to block endogenous peroxidase activity. Then the samples were washed with PBS and incubated with anti-PADI2 primary antibody (1:200) at room temperature for 1 h. Afterwards, the samples were washed with PBS again and incubated with enhanced HRP-conjugated anti-rabbit IgG secondary antibodies (ZSGB-BIO, Beijing, China) at room temperature for 20 min. Then the signals were detected by DAB reagent for 5 min and visualized by naked eye. The tissues were counterstained by Hematoxylin for 20 s. Finally, the slides were dehydrated through gradient concentrations of ethanol, incubated in xylene for 15 min and sealed using neutral balsam. Here H-score was used to analyze the expression levels of PADI2 and EMT-related markers E-cadherin and Vimentin in tumor tissues and calculated using the following formula: ${\text{H}} - {\text{SCORE}} = \Sigma \left( {P_{{\text{i}}} \, \times \,I} \right)\, \times \,{1}00$. *P*_i_ is the proportion of positive cells which was estimated from 0 to 1. Staining degree (*I*) was divided into negative, weakly positive, medium positive and strong positive, which are 0, 1, 2 and 3 respectively. 5 fields were randomly selected at high magnification.

### Statistical analysis

*P* values were calculated using one-way analysis of variance with GraphPad Prism Version 6.0. Data are expressed as means ± SD of three independent experiments. *P* < 0.05 was considered to be statistically significant difference.

## Results

### PADI2 was upregulated in ovarian cancer samples and high PADI2 expression was correlated with poor outcome

**Fig.1:**
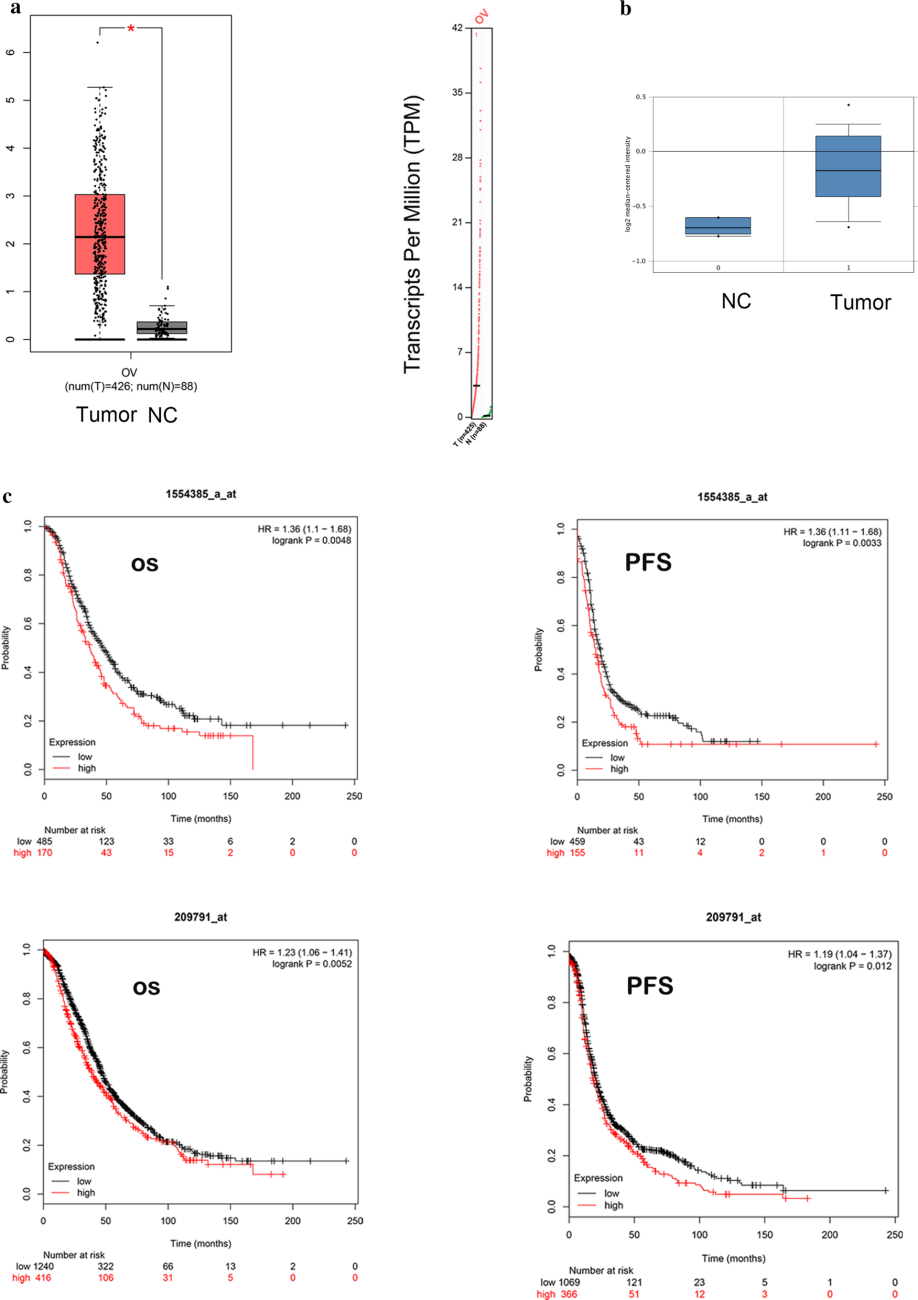
PADI2 was upregulated in ovarian cancer samples and high PADI2 expression was correlated with poor outcome. **a** Analysis of PADI2 expression level in human OC. Gene Expression Profiling Interactive Analysis (GEPIA) showed that the red and gray boxes represent cancer and normal tissues, respectively. **b** Box plots derived from gene expression data in Oncomine comparing expression of PADI2 gene in normal tissue (left plot) and ovarian cancer tissue (right plot). **c** Kaplan–Meier survival statistics analysis for the relationship between survival time and PADI2 signature in ovarian cancer was performed by using the online tool (https://kmplot.com/analysis/).*P < 0.05, **P < 0.01 and ***P < 0.001 compared with control group

To identify the gene signatures associated with PADI2 in OC, we utilized Gene Expression Profiling Interactive Analysis (GEPIA) to show the differential expression. GEPIA pictures showed that the red and gray boxes represented cancer and normal tissues, respectively. In Fig. 1a picture, 426 OC tumor samples and 88 paired non-tumor tissues were sequenced and analyzed. We found that PADI2 expression was upregulated in tumor tissues compared with nontumor tissues. Besides, the box plots derived from gene expression data in Oncomine suggested that the expression of PADI2 gene in ovarian cancer tissue was significantly increased comparing with that in normal tissue (left plot) (Fig. 1b).Furthermore, we searched the Kaplan–Meier plotter database to explore the association between the PADI2 expression level and the prognosis of OC patients. Kaplan–Meier curves for target genes were generated with the online tool Kaplan–Meier Plotter(https://www.kmplot.com/). A total of 1436 (for PFS) and 1657 (for OS) RNAseq data samples of ovarian cancer patients were split into 2 groups (high expression of PADI2 and low expression of PADI2) based on the expression level. This website accumulated 1554385_a_at and 209791_at data on the gene expression and survival of 614 and 1435 ovary cancer patients for the progression-free survival (PFS), 655 and 1656 ovary cancer patients for the overall survival (OS), respectively. We analyzed the PFS and OS of ovarian cancer patients stratified by high and low expression of PADI2 using the Kaplan–Meier curves, the log rank test and the hazard ratio with 95% confidence intervals (CI).Kaplan–Meier survival analysis showed that patients with PADI2 overexpression had significantly lower overall survival and progression-free survival than those with lower levels of PADI2 (*P* = 0.0048 for OS, 0.0033 for PFS in 1554385_a_at and 0.0052 for OS, 0.012 for PFS in 209791_at, respectively; Fig. 1c).Collectively, these results suggested that PADI2 might be an oncogene for ovarian cancer.

### To construct stable ovarian cancer cell lines A2780 and SKOV3 with low expression of PADI2

**Fig.2:**
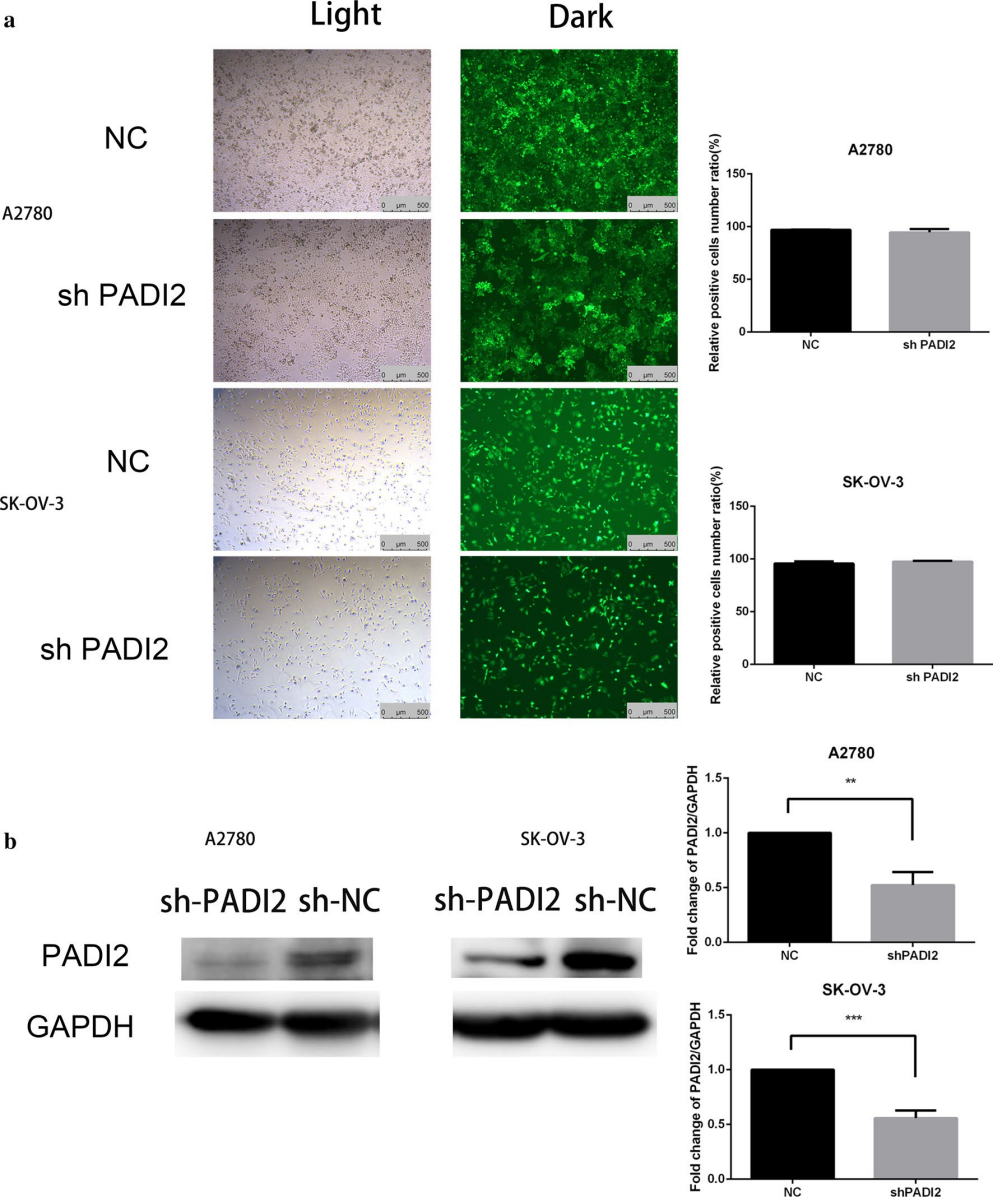
Stable knockdown of PADI2 in ovarian cancer cell lines after 72 h lentivirus infection and its effect on the expression of translator-level proteins. **a** Ovarian cancer cells in the bright field are at the left and fluorescence cells in the same field are at the right. Green fluorescence showed the cells that had been successfully transfected with lentivirus. It could be seen from the figure that infection abundance was high. The scale is 500 μm. **b** Western blot was used to verify the changes in the translation level of PADI2. GAPDH was used as the internal control. All data were expressed as the mean ± standard deviation of three repeated experiment values. *P < 0.05, **P < 0.01, ***P < 0.001

After 72 h lentivirus infection, it can be seen from Fig. 2a that the infection abundance is high in A2780 and SKOV3 ovarian cancer cells. The infection efficiency observed by fluorescence microscope is close to 100%. The cell morphology is normal and vigorous. The MOI value is 20. In order to prevent gene off-target effect, shRNA lentivirus was used to down-regulate the expression of PADI2. The infection was carried out according to the infection conditions in our previous experiment. SKOV3 cell line with low expression of PADI2 was named as SKOV3-shPADI2 cell line and A2780 cell line with low expression of PADI2 was named as A2780-shPADI2 cell line. Total protein was extracted for western blot experiment. Western blot was used to verify PADI2 changes at translation level. The statistical figure showed that PADI2 expression of lentivirus infection group was obviously lower than the control group. We found that PADI2 expression of A2780-shPADI2 and SKOV3-shPADI2 was significantly decreased to half of that in the control cells, respectively (*P* < 0.05) as shown in Fig. 2b.

### Downregulating PADI2 suppresses colony formation and proliferation of A2780 and SKOV3 cells

**Fig. 3:**
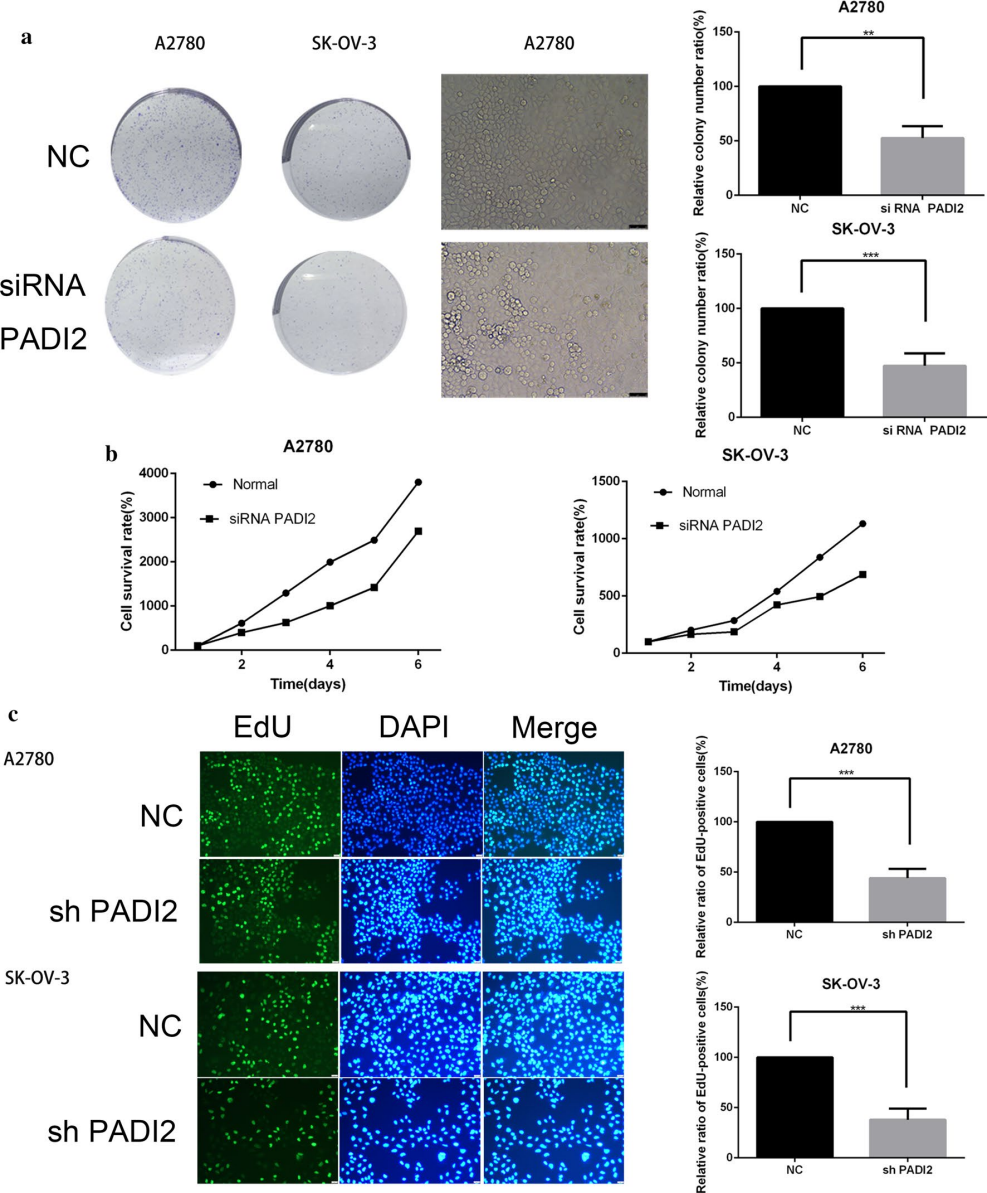
Downregulating PADI2 suppresses colony formation and proliferation of A2780 and SKOV3 cells. **a** PADI2 knockdown inhibited A2780 and SKOV3 cell number of colony formation. **b** MTT assay examined the proliferation ability of A2780 and SKOV3 cells after PADI2 knockdown. **c** EdU proliferation of A2780 and SKOV3 cells after PADI2 knockdown. A2780 and SKOV3 cells were treated as indicated and stained for EdU incorporation (green) or DAPI (blue) to highlight nuclei(Scale bar 25 μm). EdU positive A2780 and SKOV3 cells ratio was shown on the right side. All data are expressed as the mean ± SD of values from triplicate experiments. *P < 0.05, **P < 0.01, ***P < 0.001 compared with control group

To further verify the effect of PADI2 expression on the proliferation ability of A2780 and SKOV3 ovarian cancer cells, the proliferation ability of ovarian cancer cells was detected by clone formation assay, MTT assay and EdU assay. We observed the long-term inhibition of PADI2 on the proliferation of EOC cells using clone formation assay. Compared with the control group, PADI2 knockdown inhibited the proliferation of A2780 and SKOV3 cells and the colony number was significantly less than that of the control group (*P* < 0.05, Fig. 3a). Furthermore, we used MTT assay to determine the effect of PADI2 knockdown on cell viability by siRNA. The results suggested that compared with the control group, PADI2 knockdown inhibited the proliferation of A2780 and SKOV3 cells and the slope of growth curve was lower than that of the control group (*P* < 0.05, Fig. 3b). Finally, EdU is a thymidine nucleoside analogue, which enters the DNA molecule in replication when A2780 and SKOV3 ovarian cancer cells proliferate. Under the fluorescence microscope, it can give off green light, while the nucleus is stained with DAPI to give off blue light. The proliferation activity of A2780 and SKOV3 cells can be judged according to the number of green/blue fluorescence spots. Experimental results showed that compared with the control group, SKOV3 and A2780 cell lines with PADI2 knockdown significantly inhibited EdU uptake rate. In other word, their proliferation capacity was significantly inhibited, as shown in Fig. 3c. In summary, these results suggested that the level of PADI2 expression may affect the ability of SKOV3 and A2780 ovarian cancer cells to proliferate in vitro. PADI2 knockdown may be a potential anticancer approach for SKOV3 and A2780 ovarian cancer cell lines.

### Downregulating PADI2 suppresses the migration and invasion of A2780 and SKOV3 cells

**Fig.4:**
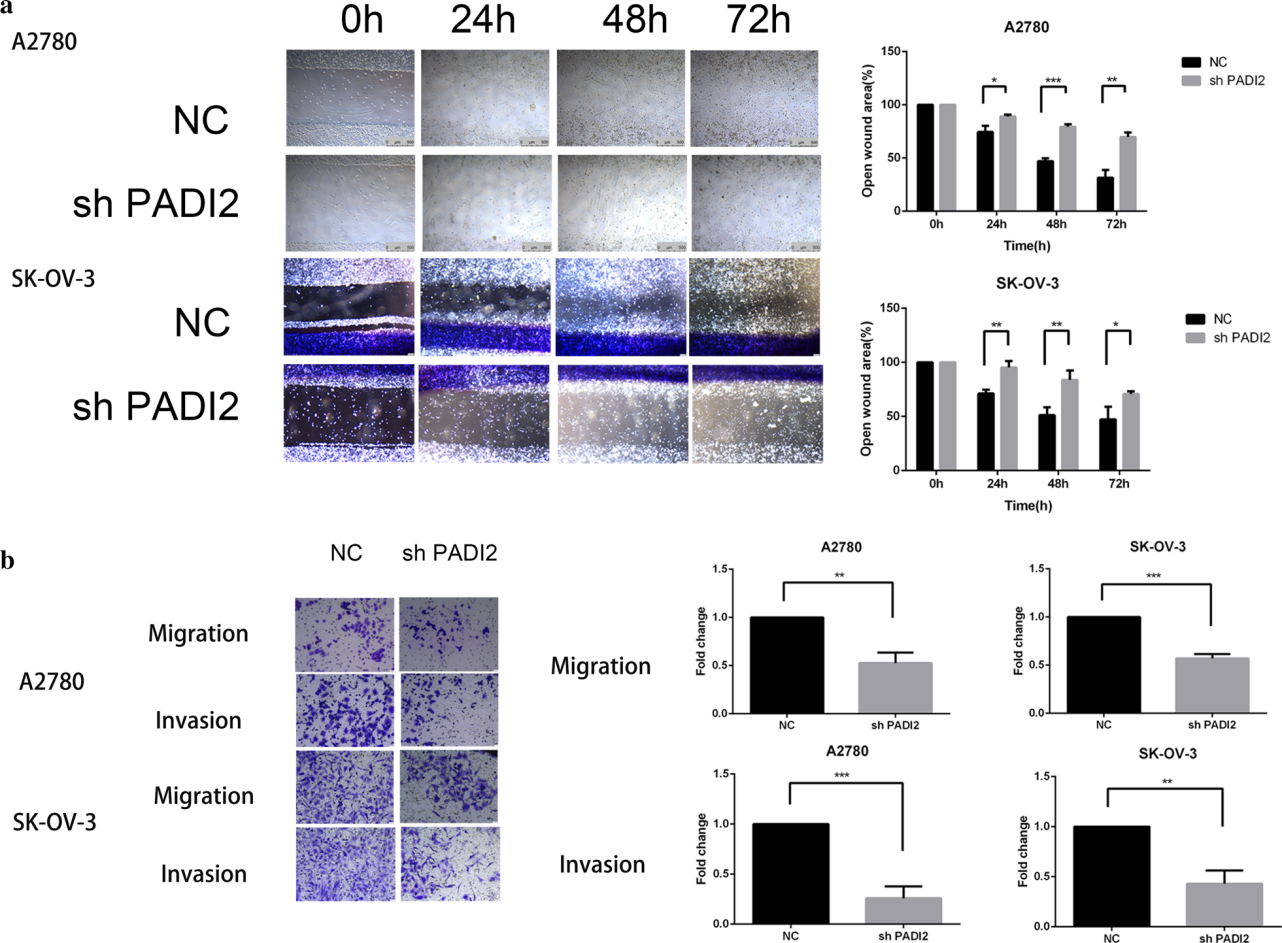
Downregulating PADI2 suppresses the migration and invasion of A2780 and SKOV3 cells. **a** Wound-healing assay was performed in A2780 and SKOV3 cells(Scale bar 100 μm). **b** Cell migration and migrated number were showed by transwell assay in A2780 and SKOV3 cells after PADI2 knockdown (Scale bar 25 μm). Quantitative analyses of migration and invasion abilities showed in the right side. All data are expressed as the mean ± SD of values from triplicate experiments. *P < 0.05, **P < 0.01 and ***P < 0.001 compared with control group

A2780 and SKOV3 cells migration ability was tested by wound healing assay. After the scratch of A2780 and SKOV3 ovarian cancer cells, the cells migrated to the scratch area were observed and photographed with an inverted microscope at 0, 24, 48 h and 72hours points. Then, the scratch areas of A2780 and SKOV3 cell lines were compared at each time point. As shown in Fig. 4a, our results showed that at the above time points, the cell migration ability of PADI2 knockdown group was significantly lower than that of control group(*P* < 0.05). We further investigated the effect of PADI2 expression on the invasiveness of A2780 and SKOV3 ovarian cancer cells in vitro by transwell assays. Matrigel was prepared at the bottom of transwell chamber to compare the differences in the number of SKOV3-shPADI2 cell lines and A2780-shPADI2 cell lines with the control cells. The results showed that the number of SKOV3-shPADI2 and A2780-shPADI2 cells crossing the transwell subventricular membrane was far less than that of the control group (*P* < 0.05), as shown in Fig. 4b. Collectively, It indicated that PADI2 knockdown could inhibit the invasiveness of A2780 and SKOV3 ovarian cancer cells.

### Downregulating PADI2 and Olaparib combination treatment attenuated the viability of ovarian cancer cells

**Fig. 5:**
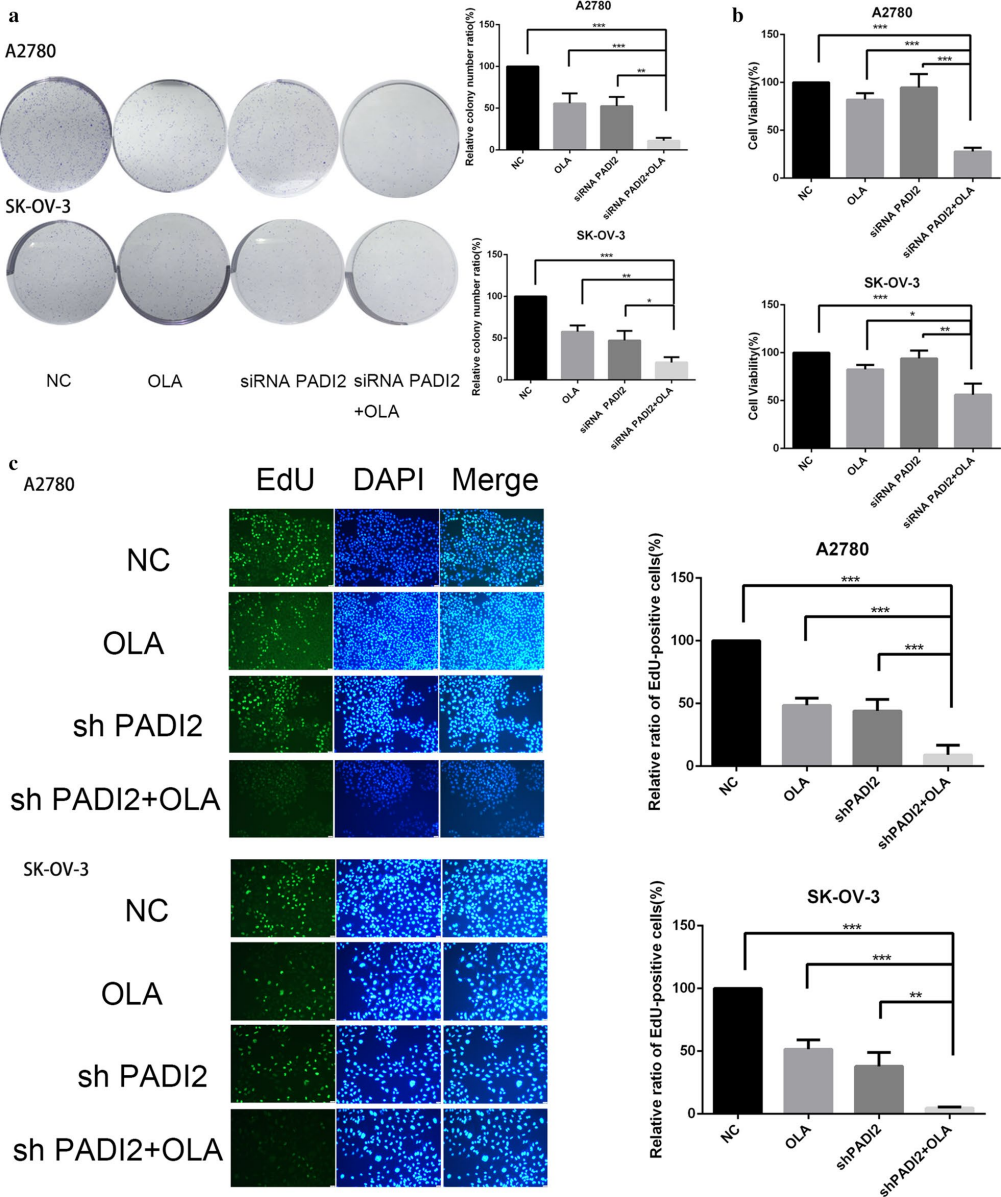
Downregulating PADI2 and Olaparib combination treatment attenuated the viability of ovarian cancer cells. **a** PADI2 knockdown inhibited A2780 and SKOV3 cell number of colony formation with and without the treatment with Olaparib. **b** MTT assay examined the proliferation ability of A2780 and SKOV3 cells after PADI2 knockdown with and without the treatment with Olaparib. **c** EdU proliferation of A2780 and SKOV3 cells after PADI2 knockdown with and without the treatment with Olaparib. A2780 and SKOV3 cells were treated as indicated and stained for EdU incorporation (green) or DAPI (blue) to highlight nuclei(Scale bar 25 μm). EdU positive A2780 and SKOV3 cells ratio was shown on the right side. All data are expressed as the mean ± SD of values from triplicate experiments. *P < 0.05, **P < 0.01 and ***P < 0.001 compared with control group

To confirm the role of PADI2 on anti-cancer effect, we silenced the expression of PADI2 and examined the modulation of PADI2 expression by Olaparib treatment combined with PADI2 knockdown in SKOV3 and A2780 ovarian cancer cells. As demonstrated in Fig. 5a, the colony formation assay showed that transfection with PADI2 siRNA made inhibitory effect on EOC cell proliferation. Colony numbers of PADI2 knockdown A2780 and SKOV3 cells combined with Olaparib were significantly fewer than cells treated with Olaparib alone in A2780 and SKOV3 ovarian cancer cell lines.(*P* < 0.05, Fig. 5a). Furthermore, the cell viability was estimated by using the MTT assay in A2780 and SKOV3 ovarian cancer cell lines, as shown in Fig. 5b. Additionally, we used EdU staining assay to find that knockdown of PADI2 combined with Olaparib treatment significantly inhibited EdU uptake rate in comparison to cells treated with Olaparib alone in A2780 and SKOV-3 cells (Fig. 5c). Collectively, these findings suggested that downregulation of PADI2 enhanced the sensitivity of SKOV3 and A2780 cells toward Olaparib treatment.

### Down-regulation of PADI2 and Olaparib combination treatment suppresses the migration and invasion of A2780 and SKOV3 cells

**Fig.6:**
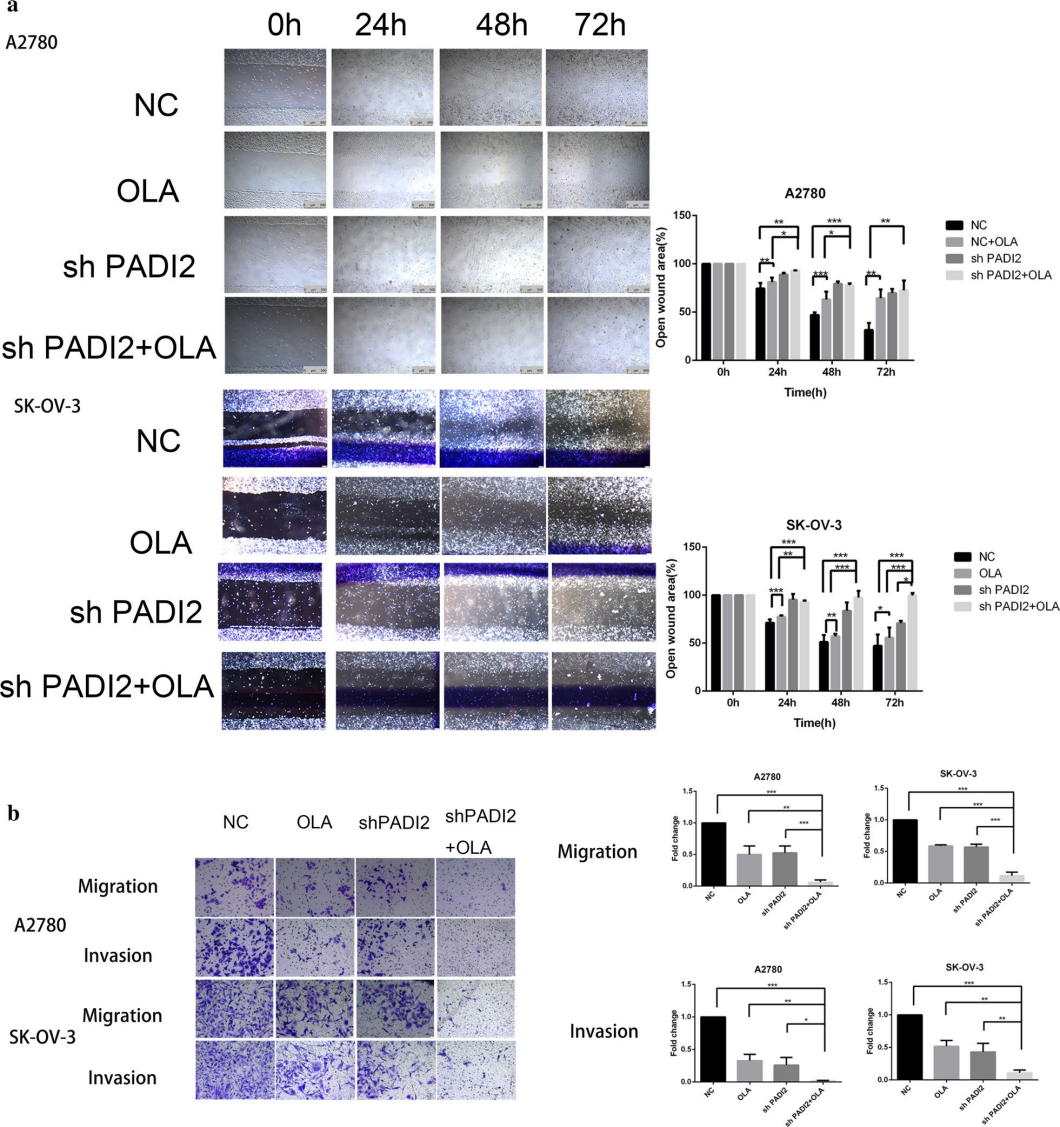
Downregulating PADI2 and Olaparib combination treatment suppresses the migration and invasion of A2780 and SKOV3 cells. **a** Wound-healing assay was performed in A2780 and SKOV3 cells(Scale bar 100 μm). **b** Cell migration and migrated number were showed by transwell assay in A2780 and SKOV3 cells after PADI2 knockdown with and without the treatment with Olaparib(Scale bar 25 μm). Quantitative analyses of migration and invasion abilities showed in the right side. All data are expressed as the mean ± SD of values from triplicate experiments. *P < 0.05, **P < 0.01 and ***P < 0.001 compared with control group

A2780 and SKOV3 cells migration ability was tested by wound healing assay. After the scratch of A2780 and SKOV3 ovarian cancer cells, the cells migrated to the scratch area were observed and photographed with an inverted microscope at 0, 24, 48 and 72 h points. Then, the scratch areas of A2780 and SKOV3 cell lines were compared at each time point. As shown in Fig. 6a, our results showed that at the above time points, the cell migration ability of PADI2 knockdown and Olaparib treatment group was significantly lower than that of Olaparib treatment group alone (*P* < 0.05). Compared with the control group, the migration ability of SKOV3-shPADI2 cell line was significantly decreased, while the migration ability of A2780-shPADI2 cell line was only decreased at 24 and 48 h (*P* < 0.05). Furthermore, the effect of PADI2 on the migration and invasion capacity of cells was measured with transwell assays. To verify whether PADI2 knockdown enhanced Olaparib's anticancer effect by inhibiting the invasion ability of A2780 and SKOV3 ovarian cancer cells, matrigel was prepared at the bottom of transwell chamber to compare the differences in the number of low PADI2 expression SKOV3-shPADI2 cell lines and A2780-shPADI2 cell lines with the control cells. The results showed that the number of PADI2 knockdown and Olaparib treatment group in SKOV3-shPADI2 and A2780-shPADI2 cells crossing the transwell subventricular membrane was far less than that of Olaparib treatment group alone (*P* < 0.05), as shown in Fig. 6b. It indicated that PADI2 knockdown could inhibit the invasiveness of A2780 and SKOV3 ovarian cancer cells and enhanced the ability of Olaparib to inhibit the invasiveness of ovarian cancer cells. Overall, these results suggest that down-regulation of PADI2 enhanced Olaparib's ability to inhibit invasion and migration of A2780 and SKOV3 ovarian cancer cells.

### Identification of differentially expressed genes (DEGs) in A2780-shPADI2 and SKOV3-shPADI2 cell by transcriptome sequencing analysis

**Fig.7:**
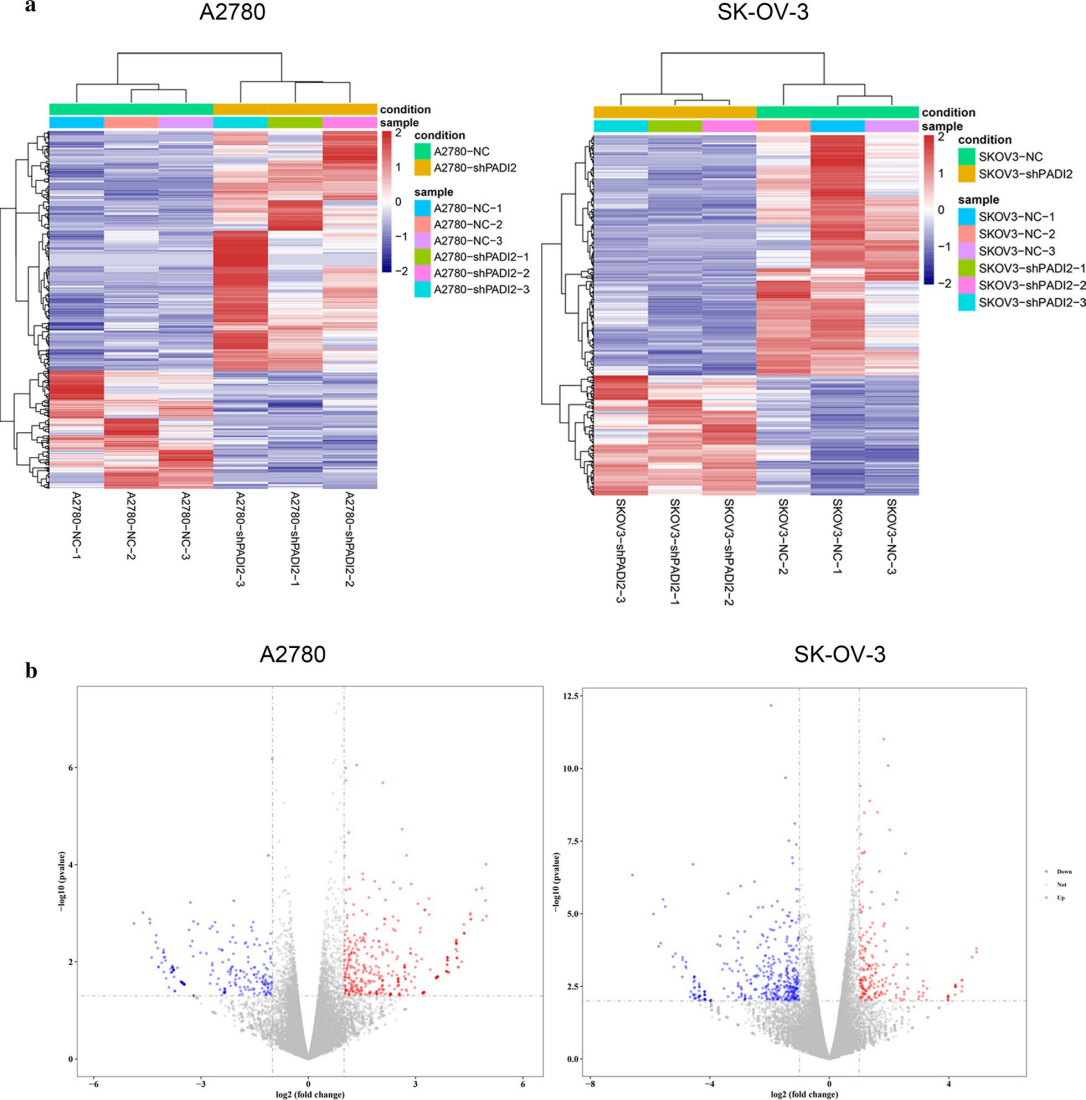
Identification of differentially expressed genes (DEGs) in A2780-shPADI2 and SKOV3-shPADI2 cell by transcriptome sequencing analysis. **a** Clustering heatmap of the 22,618 and 22,995 genes exhibiting significantly differential expression, statistically significant DEGs were defined as |Log2 Fold change|> 2 and *P* value < 0.05 in A2780-shPADI2 and SKOV3-shPADI2 cell than A2780 and SKOV3 cell, respectively. **b** Volcano plot of the 22,618 and 22,995 expressed genes in A2780-shPADI2 and SKOV3-shPADI2 cell than A2780 and SKOV3 cell, respectively. Red color represented up-regulated genes in A2780-shPADI2 and SKOV3-shPADI2 cell than A2780 and SKOV3 cell and blue color represented down-regulated genes. All data are expressed from triplicate experiments

The transcriptome sequencing results showed that PADI2 knockdown ovarian cancer cell line A2780-shPADI2 had a difference on the condition of |Log2 Fold change|> 2 and *P*-value < 0.05. Clustering heatmap of the 22,618 and 22,995 genes exhibiting significantly differential expression. There were 200 differential genes, including 55 down-regulated genes and 145 up-regulated genes. There were 625 differentially expressed genes in SKOV3-shPADI2 cell lines, including 417 down-regulated genes and 208 up-regulated genes. The cluster analysis of heat map showed the differentially expressed genes that had good repeatability (Fig. 7). Red color represented up-regulated genes in A2780-shPADI2 and SKOV3-shPADI2 cell than A2780 and SKOV3 cell and blue color represented down-regulated genes. Volcano plot showed the 22,618 and 22,995 expressed genes in A2780-shPADI2 and SKOV3-shPADI2 cell than A2780 and SKOV3 cell, respectively.

### Downregulating PADI2 and Olaparib combination treatment suppresses EMT and JAK2/STAT3 signaling pathway in A2780 and SKOV3 cells

**Fig.8:**
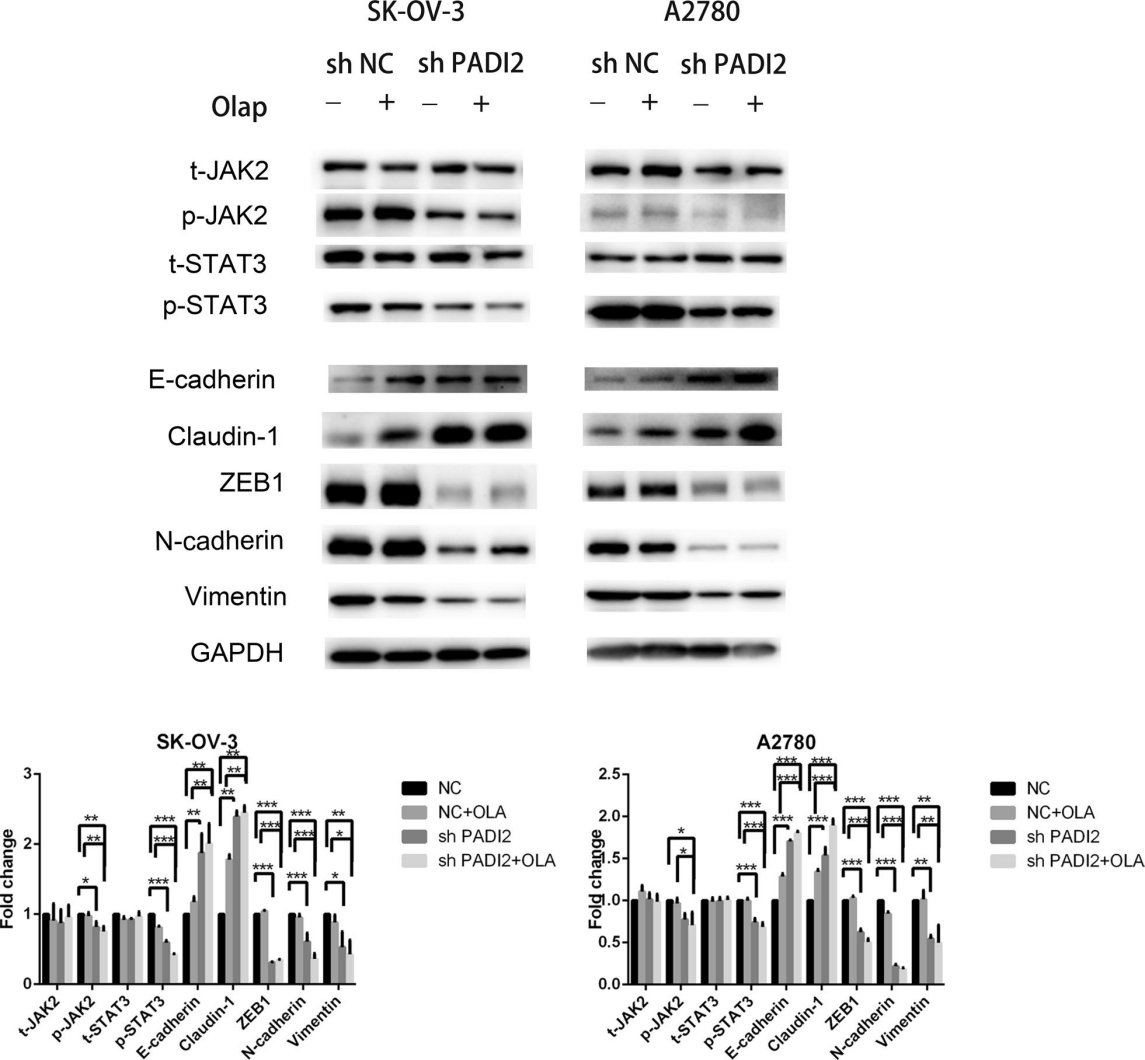
Downregulating PADI2 and Olaparib combination treatment suppresses EMT and JAK2/STAT3 signaling pathway in A2780 and SKOV3 cells. Western blotting analyses of SKOV3 and A2780 cells treated as described above. E-Cadherin, Claudin-1, Vimentin, ZEB1, N-Cadherin, phospho-Stat3 (Tyr705), phospho-Jak2 (Tyr1007), t-Stat3 and t-Jak2 levels were carried out. GAPDH served as a loading control. Fold changes of the proteins were shown on the bottom. All data are expressed as the mean ± SD of values from triplicate experiments. *P < 0.05, **P < 0.01 and ***P < 0.001 compared with control group

Through the screen of differential genes and bioinformatics analysis, genes were closely related to ovarian cancer after downregulation of PADI2, such as STAT3 which was identified for further in-depth study on the occurrence and development of ovarian cancer. Therefore, we hypothesized that STAT3 phosphorylation might be related to the biological behavior of PADI2-mediated ovarian cancer cells. We verified the sequencing results of STAT3 phosphorylation levels in A2780 and SKOV3 ovarian cancer cells by western blot. As shown in Fig. 8, compared with A2780 and SKOV3 cells treated with Olaparib alone, the phosphorylation of JAK2 and STAT3 in cells treated with PADI2 and Olaparib was decreased, while the total amount of JAK and STAT3 of remained unchanged. Phosphorylation of STAT3 is not due to a reduction in total STAT3 expression. JAK2 is an upstream kinase that activates and phosphorylates STAT3. Furthermore, western blot analysis showed that the expressions of EMT-related protein molecules in PADI2 knockdown combinated with Olaparib group were decreased, compared with A2780 cells and SKOV3 cells treated with Olaparib alone. The expressions of epithelial phenotype such as E-Cadherin and claudin-1 were up-regulated, while the expressions of mesenchymal phenotypes such as Vimentin, ZEB1 and N-Cadherin were down-regulated. The above experimental results suggested that PADI2-mediated phosphorylation of STAT3 was associated with EMT and JAK2/STAT3 signaling pathway in A2780 and SKOV3 ovarian cancer cell lines.

### Downregulation of PADI2 combinated with Olaparib repressed the proliferation of tumor cells in vivo

**Fig.9:**
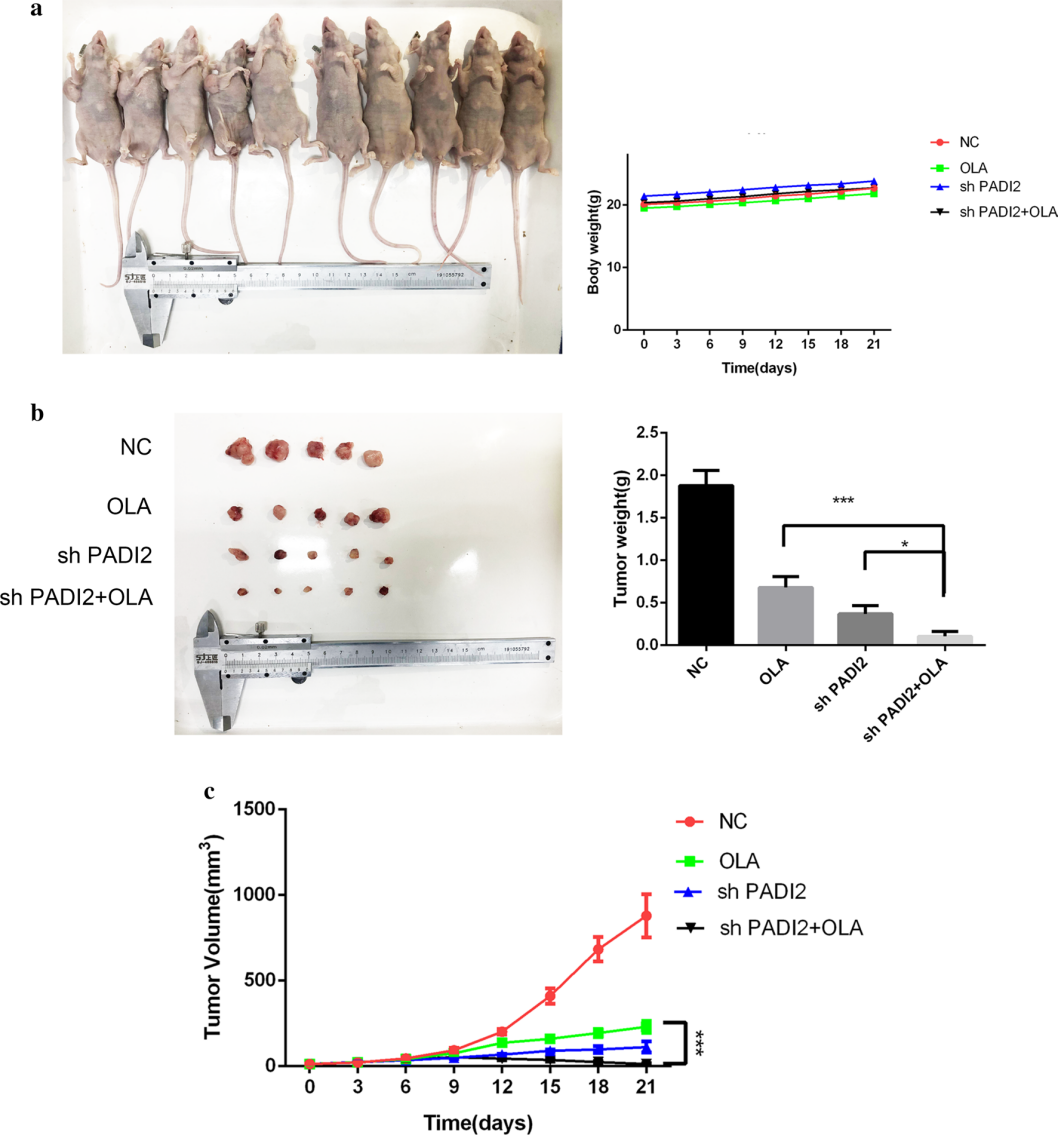
Downregulation of PADI2 combinated with Olaparib repressed the proliferation of tumor cells in vivo. **a** SKOV3 stable cell line was used to construct ovarian cancer subcutaneous xenograft model of nude mouse. The body weight of each mouse was measured every three days since the indicated treatments began. n = 5. **b** The nodules were lighter in SKOV3-shPADI2 combinated with Olaparib group compared with the control group. Quantification of tumors weight is on the right. **c** Tumors grew more slowly after transfection of PADI2 shRNA in vivo. Quantitative analyses of tumors growth. *P < 0.05, **P < 0.01, ***P < 0.001

After 25 days of SKOV3 cells reaching the subcutaneous surface of nude mice, nodules with a diameter of about 3 mm could be reached at the inoculation site, with a tumor formation rate of 100% and a tough texture. The tumor-bearing nude mice were generally in good condition. They were randomly assigned to receive intraperitoneal injection of Olaparib for 21 days and were well tolerated, as shown in the Fig. 9a. There were no obvious abnormalities in diet and activity and no obvious adverse reactions. Tumor growth curve showed that the growth of subcutaneous transplantation tumor of shPADI2 group was obviously restrained from 10th day and tumor volume of shPADI2 group was significantly less than control group on average at the end of Olaparib treatment. The mean weight and average volume of tumors in the down-regulated PADI2 combined with Olaparib group were significantly lower than that in the group with Olaparib alone.

### Downregulation of PADI2 combinated with Olaparib inhibited epithelial-mesenchymal transition and STAT3 of tumor in vivo

**Fig. 10:**
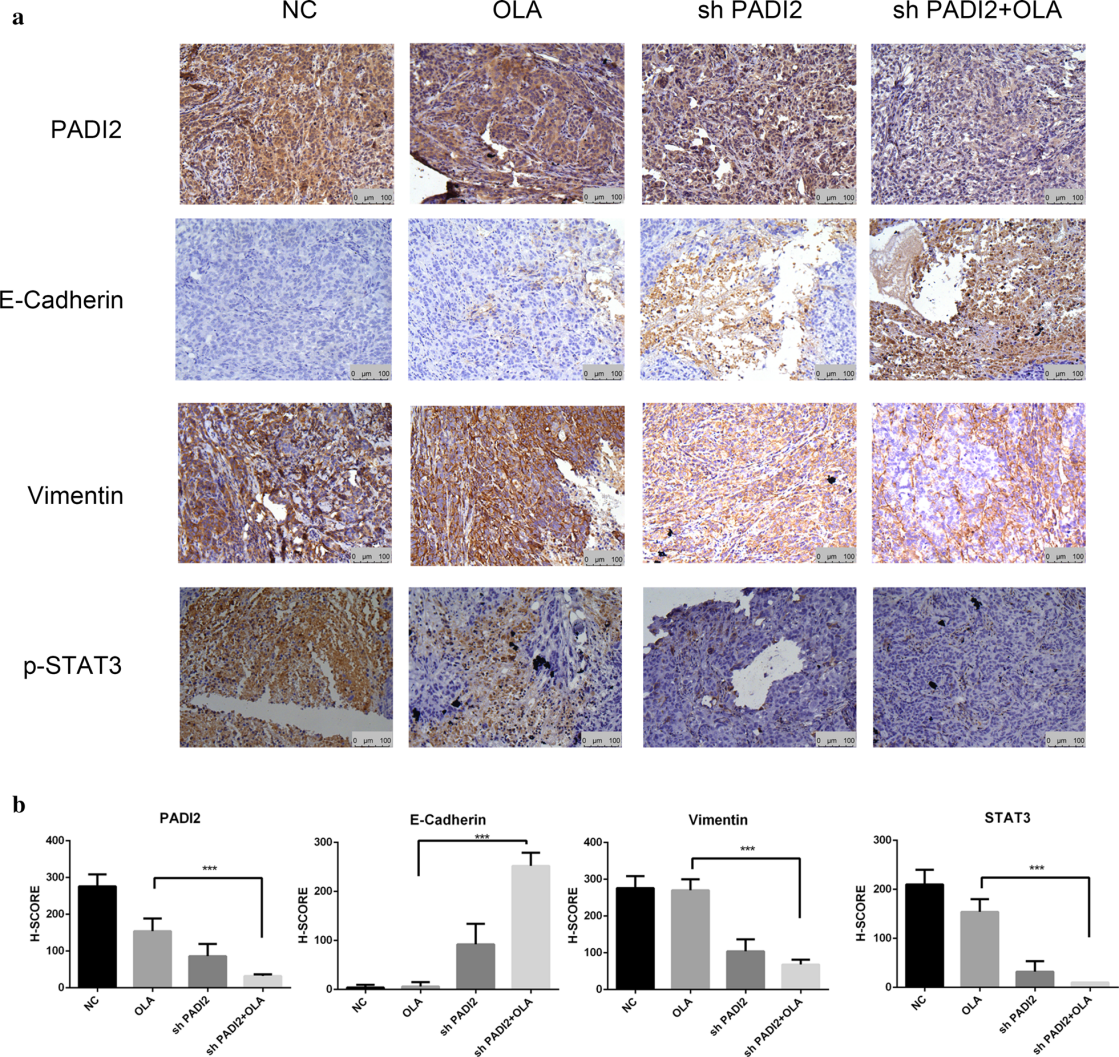
Downregulation of PADI2 combinated with Olaparib inhibited epithelial-mesenchymal transition and STAT3 of tumor in vivo. **a** Representative IHC staining showed the expression of PADI2 E-cadherin, Vimentin and phospho-STAT3 in tumor issues (× 200 magnification, scale:100 μm). **b** Quantification of the expression of PADI2 E-cadherin, Vimentin and phospho-STAT3. *P < 0.05, **P < 0.01, ***P < 0.001

Immunohistochemical staining results showed that E-Cadherin expression level of down-regulated PADI2 combined with Olaparib group was significantly higher than that in the group with Olaparib alone. The expression of PADI2, Vimentin and p-STAT3 level of down-regulated PADI2 combined with Olaparib group was significantly lower than that in the group with Olaparib alone, as shown in Fig. 10. Our results suggested that down-regulation of PADI2 combined with Olaparib played the role of anti-ovarian cancer by inhibiting EMT and STAT3 signaling pathway.

## Discussion

Epithelial ovarian cancer (EOC) is the most fatal disease among malignant tumors of the female reproductive system. In the present study, we investigated the role of PADI2 on EOC in vitro and in vivo for the first time. PADI2 was upregulated in ovarian cancer samples and high PADI2 expression was correlated with poor outcome. After we constructed stable ovarian cancer cell lines A2780 and SKOV3 with low expression of PADI2, Downregulating PADI2 suppresses colony formation, proliferation, migration and invasion of A2780 and SKOV3 cells. Furthermore, downregulating PADI2 and Olaparib combination treatment attenuated the viability, migration and invasion of A2780 and SKOV3 cells. We identified differentially expressed genes (DEGs) in A2780-shPADI2 and SKOV3-shPADI2 cell by transcriptome sequencing analysis, verified that downregulating PADI2 and Olaparib combination treatment suppresses EMT and JAK2/STAT3 signaling pathway in A2780 and SKOV3 cells in vitro and in vivo.

Olaparib is an inhibitor of the Poly-ADP-ribose polymerase (PARP) family, which plays an important role in the DNA repair pathway and contains 17 proteases [33]. DNA repair was performed by binding ADP ribose (PAR) portion of the zinc finger domain to the single-stranded DNA damage site [34]. A recent study [35] showed that the PFS of BRCA mutation in platinum-sensitive patients with recurrent ovarian cancer was significantly prolonged in SOLO2 study by the European Network for Gynecological Oncological Trial (ENGOT) Groups. Although the emergence of Olaparib is a breakthrough in the maintenance therapy of ovarian cancer, drug resistance has also emerged [4, 5]. Several studies have shown that PADI2 enhances carcinogenesis in a variety of cancers, including breast cancer, spontaneous skin tumors, epithelial bladder cancer, and colon cancer. Our previous results showed that the expression of PADI2 was significantly increased in immunohistochemistry of a variety of cancers, including serous ovarian papillary adenocarcinoma, compared to normal tissues. In this study, we determined that PADI2 was highly expressed in ovarian cancer samples and associated with poor prognosis. We built two stable PADI2 low expression of ovarian cancer cell lines for subsequent experiment and verified the effect of PADI2 expression on the proliferation of A2780 and SKOV3 cells.

Cantarino et al. [36] found that down-regulation of PADI2 was an early event in the pathogenesis of colorectal cancer and associated with poor prognosis, suggesting the tissue specific role of PADI2 in tumor progression. The expression of PADI2 gene in colon cancer has been shown to be down-regulated and PADI2 inhibits the proliferation of colon cancer cells [18]. However, McElwee et al. [19] pointed out that the expression of PADI2 increased during the transformation of benign breast epithelium into malignant breast cancer. Overexpression of PADI2 in transgenic mice promotes the development of skin tumors by enhancing the inflammatory response in the tumor microenvironment [20]. Sunish Mohanan et al. [16] found that the total number of dermal papilloma in PADI2 over expressed mice increased significantly compared to the control group. Above studies supported the involvement of PADI2 in tumorigenic processes in some tumors [11]. Our results are consistent with these studies, and PADI2 expression is significantly increased in ovarian cancer samples.

Epithelial mesenchymal transformation (EMT) is a process that polar epithelial cells are transformed into mesenchymal cells which acquire the ability to invade and migrate [36]. EMT is closely related to drug resistance to tumor metastasis. Chen et al. [19] reported that PADI2 was closely related to the process of EMT. Cherrington et al. [11] found that the down-regulation of PADI2 inhibited the migration of breast cancer cells and promoted the transformation of mesenchymal cells to epithelial cells. Chowdhury [23] reported three different human cancer cells, U2OS human osteosarcoma cell line, A549 human non-small cell lung cancer cell line and HeLa human cervical cancer cell line, were irradiated by gamma ray combined with Olaparib. The expression of some protein markers which involved in the cell migration and epithelial-mesenchymal transformation were changed. Olaparib treatment alone decreased cell migration and the expression of EMT pathway protein markers such as N-Cadherin and Vimentin. In his another report [24], two non-small cell lung cancer cells, A549 and p53-deficient H1299 cells, were irradiated with ^12^C and inhibited by PARP-1 to reduce cell proliferation and cell migration, respectively. Single treatment of them reduced N-Cadherin and Vimentin, but increased claudin-1 and 2 to inhibit EMT. Schacke et al. [25] reported that olaparib prevented the occurrence of EMT. McElwee [20] reported that overexpression of PADI2 in mice led to occur EMT in tumor cells, which decreased E-Cadherin expression and increased Snail and Vimentin expression. Flag-PADI2 was stably overexpressed in A431 human squamous-cell carcinoma cell line. It was found that cells that overexpressed PADI2 in vitro were more tumorigenic and elevated inflammation and EMT markers, which promoted tumor progression. Consistent with these previous studies, transcriptome sequencing and western blot analysis in our study showed that compared with A2780 cells and SKOV3 cells treated with olaparib alone, the expressions of EMT-related protein molecules were decreased in cells treated with combination of PADI2 knockdown and Olaparib. The expression of epithelial phenotypes E-Cadherin and claudin-1 were up-regulated and the expression of mesenchymal phenotypes Vimentin, ZEB1 and N-Cadherin were down-regulated. Down-regulation of PADI2 enhanced olaparib's ability to inhibit invasion and migration of SKOV3 and A2780 ovarian cancer cells by inhibiting EMT.

JAK2/ STAT3 signaling pathway regulates cell development, differentiation, proliferation, and apoptosis, which not only participates in regulating normal physiological processes, but also plays an important role in the occurrence and development of tumors [20, 37]. Multiple reports [29, 30] have shown that Olaparib is associated with JAK2 and STAT3 genes and inhibits STAT3 phosphorylation. The expression of PADI2 was up-regulated in NNK [4-(methylnitrosamino)-1-(3-pyridyl)-1butanone]-treated lung tissues and played a substantial role in the induction of lung cancer through STAT3 pathway [31]. Now it has been reported [16] that targeting PADI2 in cancer is associated with phosphorylated STAT3. Consistent with the previous results, our research showed that JAK2/STAT3 signaling pathway could be significantly inhibited after targeted downregulation of PADI2. Downregulation of PADI2 in SKOV3 and A2780 ovarian cancer cells inhibited EMT-related markers and STAT3 phosphorylation. However, the upstream of PADI2 and the role of PADI2 in olaparib against ovarian cancer cell metastasis remain to be further explored.

## Conclusion

we found that PADI2 has a tumorigenic role in EOC and a molecular basis for further studies has been provided. It has been shown that downregulation of PADI2 could suppress the proliferation, migration and invasion abilities of EOC in vitro and in vivo. Furthermore, downregulating PADI2 and Olaparib combination treatment attenuated the migration and invasion of A2780 and SKOV3 cells by inhibiting the EMT through JAK2/STAT3 signaling pathway. The findings presented here suggest a novel therapeutic approach to the Olaparib resistance of ovarian cancer and PADI2 as a prognostic marker in advanced ovarian cancer enhances sensitivity of Olaparib to ovarian cancer.

## Data Availability

All datasets used and/or analyzed during the current study are available from the corresponding author on reasonable request.
